# Severe pulmonary regurgitation in adolescents with tetralogy of Fallot leads to increased longitudinal strain

**DOI:** 10.1007/s10334-019-00780-0

**Published:** 2019-10-03

**Authors:** Pekka Ylitalo, Lauri Lehmonen, Kirsi Lauerma, Miia Holmström, Olli Pitkänen-Argillander, Eero Jokinen

**Affiliations:** 1 Children’s Hospital, University of Helsinki and Helsinki University Hospital, Helsinki, Finland; 2 HUS Medical Imaging Center, Radiology, University of Helsinki and Helsinki University Hospital, Helsinki, Finland; 3 Department of Physics, University of Helsinki, Helsinki, Finland

**Keywords:** Tetralogy of Fallot, Adolescent, Cardiovascular magnetic resonance, Feature tracking, Strain

## Abstract

**Objectives:**

Postoperative patients with tetralogy of Fallot (TOF) are often compromised by chronic pulmonary regurgitation and chronic right ventricular volume load. We sought to determine whether pulmonary regurgitation (PR) would affect right and left ventricle (RV and LV) strain.

**Materials and methods:**

This cross-sectional analysis included 40 patients who had TOF with surgical repair, with an average follow-up period of 11.8 ± 3.0 years. Altogether, 44 healthy volunteers with similar age and gender distribution were recruited. A cardiovascular magnetic resonance imaging study with feature tracking analysis was performed on all patients and controls.

**Results:**

RV peak longitudinal strain was increased in TOF patients with PR > 30 ml/m^2^ when compared to those with PR < 30 ml/m^2^ (− 22.5% ± 2.7% vs − 19.7% ± 3.5%, *p* = 0.018) and controls (*p* = 0.007). PR volume correlated with peak RV longitudinal strain (*R* = − 0.37, *p* = 0.030) and peak RV longitudinal strain rate (systolic: *R* = 0.37, *p* = 0.03; diastolic: *R* = 0.39, *p* = 0.021). The peak RV circumferential strain, from base to apex, increased more than in healthy controls (apex-base difference 7.6% ± 4.2% vs 3.3% ± 2.4%, *p* < 0.0001).

**Conclusions:**

Pediatric patients with TOF and a severe pulmonary regurgitation show an enhanced longitudinal strain when compared to patients with milder regurgitation or to control subjects. In addition, mean RV circumferential strain of the patients is significantly enhanced compared to healthy individuals.

## Introduction

Tetralogy of Fallot (TOF) is the most common cyanotic congenital heart defect. It was first successfully repaired in 1955 [1]. Before the era of corrective surgery, only half of TOF patients survived the first years of life and only a few reached adulthood [2]. However, corrective surgery has yielded good outcomes, and thus patients have improved dramatically over decades [3–5]. Decreased operative mortality has resulted in an increasing number of adolescents and adults with this congenital defect.

Even if the right ventricular outflow tract (RVOT) obstruction has been surgically repaired, the operation often results in pulmonary regurgitation and chronic right ventricular volume load [6, 7]. This pathological mechanism is considered important for the development of the long-term adverse effects observed in TOF patients [8]. In follow-up and surveillance, these patients may additionally demonstrate residual RVOT disease, right ventricular (RV) dilation or dysfunction, heart failure with left ventricular dysfunction, progressive exercise intolerance, arrhythmia, and sudden cardiac death [9–12]. These conditions may be due to the impact of electromechanical asynchrony after patching of the ventricular septal defect, shared myocardial structures, or septal deviation. In addition, recent findings of altered LV preloading due to impaired transpulmonary flow may be important for the long-term postoperative health [13–17].

Cardiovascular magnetic resonance (CMR) imaging enables accurate visualization of cardiac morphology and motion tracking through quantitative methods. Strain analysis is a widely used principle for quantification of left-ventricular function as strain describes the shortening, thickening, and lengthening of myocardial muscle. In CMR, strain can be measured with myocardial tagging and feature tracking (FT). Recently, CMR was described as the gold standard in the analysis of RV function in repaired TOF [18]. CMR FT enables retrospective strain analysis from cine images used for conventional volumetric analysis. Previous FT studies of pediatric patients with TOF have focused on dyssynchrony assessment, incremental prognostic value of right ventricular longitudinal strain, and ventricular strain parameter correlation [19–21]. In the present study, we utilized CMR feature tracking in the analysis of systolic and diastolic strain parameters of 40 children with repaired TOF and compared the results with those of 44 healthy children. We hypothesized that since the basal and outlet regions of the RV are injured at repair, possible compensatory changes in myocardial function may take place within the intact part of the RV.

## Materials and methods

### Study description

This prospective single-center observational study included 45 patients who had Tetralogy of Fallot (20 girls and 25 boys). The surgical repair of TOF had been performed in these patients between 1990 and 2003. The patients were referred to CMR by a pediatric cardiologist, because their echocardiographic findings suggested a significant pulmonary regurgitation and increased RV size. Three patients declined from the study and two were rejected because of insufficient CMR image quality. Thus, 40 were admitted to the study and were examined during an annual ambulatory visit to the clinic.

The patients had undergone surgical correction for tetralogy of Fallot at the age of 1.2 ± 1.1 years. The postoperative follow-up period was 11.8 ± 3.0 years. Transannular patch was used in 20 (50%) patients, and pulmonary valve annulus could be preserved at the primary repair in 20 patients. Prior to correction three patients had received a palliative shunt. At repair, cardiopulmonary bypass with aortic crossclamp was used. Myocardial protection was achieved with cold blood cardioplegia. We observed no time-related trends in surgical techniques used in our patients’ operations from 1990 to 2003.

Forty-four healthy pediatric and adolescent volunteers with similar age and gender distribution were recruited for control subjects. The admission criteria were: (i) no medical history of any cardiovascular disease and (ii) no other pre-existing condition that would affect the cardiovascular system. A pediatric cardiologist performed a morphologic ultrasound and an ECG was recorded to exclude possible latent cardiac problems. The characteristics of the study population and control subjects are presented in Table 1. None of the patients had significant tricuspid regurgitation, and thus, the RV afterload was estimated to be normal in the echocardiography*.*

**Table 1:** Baseline characteristics of the study population and healthy controls

Parameter	Healthy (*N* = 44)	TOF (*N* = 40)	*p* value	TOF PR < 30 ml/m^2^ (*N* = 29)	TOF PR > 30 ml/m^2^ (*N* = 11)	*p* value
Number of females *N* (%)	17 (39)	15 (38)	–	12 (41)	3 (27)	–
Age at TOF repair (years)	–	1.2 ± 1.1	–	1.3 ± 1.2	1.1 ± 0.8	0.583
Follow-up time from repair (years)	–	11.8 ± 3.0	–	11.9 ± 2.7	11.5 ± 3.7	0.734
Age at CMR examination (years)	14.1 ± 3.4	13.0 ± 3.3	0.117	13.2 ± 3.2	12.5 ± 3.7	0.612
Height (cm)	160.8 ± 16.0	152.2 ± 16.1	0.15	153.8 ± 16.3	147.6 ± 15.1	0.269
BSA (m^2^)	1.5 ± 0.3	1.3 ± 0.3	0.009*	1.4 ± 0.3	1.3 ± 0.3	0.507
HR (bpm)	68.1 ± 12.4	79.0 ± 15.8	0.001*	78.9 ± 15.2	79.1 ± 17.3	0.973
Pulmonary regurgitation (ml/m^2^)	1.3 ± 4.7	19.8 ± 14.3	< 0.0001*	13.2 ± 9.9	37.1 ± 8.4	< 0.0001*
RVEDV (ml/m^2^)	102.9 ± 17.5	131.0 ± 23.4	< 0.0001*	124.8 ± 21.2	147.2 ± 22.0	0.01*
RV EF (%)	59.5 ± 6.3	54.5 ± 6.5	0.001*	53.7 ± 6.1	56.5 ± 7.4	0.272
LVEDV (ml/m^2^)	94.9 ± 16.6	88.9 ± 12.8	0.066	91.7 ± 13.4	81.5 ± 7.4	0.005*
LV EF (%)	63.3 ± 6.8	58.0 ± 6.3	0.0003*	59.0 ± 6.8	55.5 ± 3.7	0.045*

*TOF* repaired tetralogy of Fallot, *PR* pulmonary regurgitation, *CMR* cardiovascular magnetic resonance, *BSA* body surface area, *RVEDV* right ventricle end-diastolic volume

*Statistically significant difference (*p* value < 0.05)

Our study protocol was validated and approved by the Ethics Committee of the Children’s Hospital of the Helsinki University Hospital. We obtained a written informed consent from all the participants and/or their parents.

### Imaging protocol and volumetric analysis

CMR was performed on all patients and controls. CMR images were obtained with a 1.5 T Achieva system (Philips Healthcare, The Netherlands) using a 5-channel cardiac coil. For right-ventricular-volume analysis, transaxial and left-ventricle short axis balanced turbo field echo breath-hold cine images were obtained throughout the whole heart. Slice thickness was 5–8 mm, depending on the patient’s size, gap was 20%, and temporal resolution was 26–43 ms. Cine images had 30 temporal phases in the short-axis direction and 25 temporal phases in the long-axis direction. For the ventricular volume measurements, both the transaxial and the LV short axis cine images were manually planimetered with the cardiac analysis tool of a Philips ViewForum workstation (Philips Healthcare, The Netherlands). Pulmonary-valve regurgitation fraction was calculated from volumetric measurements of both ventricles. Two radiologists experienced in CMR performed all ventricular CMR analyses.

### Feature tracking analysis

**Fig. 1:**
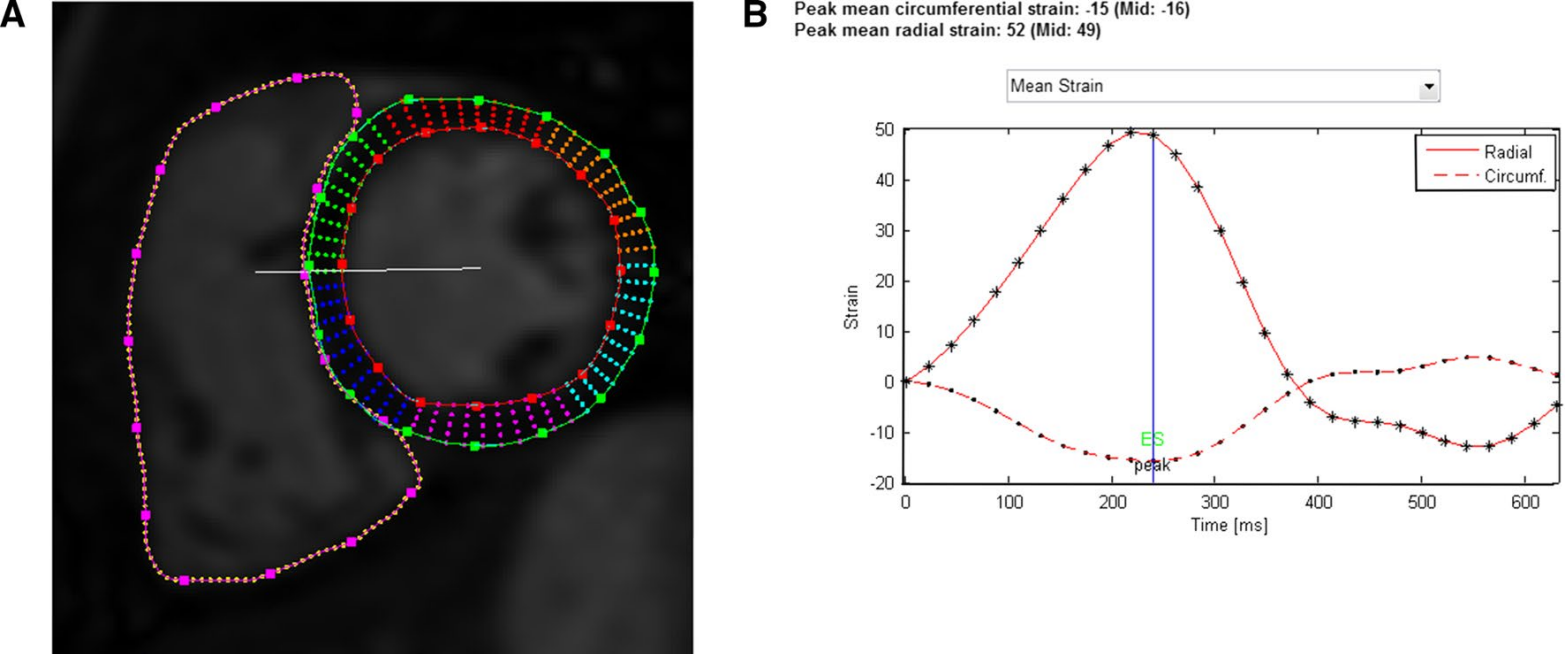
**a** Mid-ventricular short-axis view of the left and right ventricles in a patient with TOF at end diastole. LV epicardial and endocardial borders as well as RV endocardial borders have been manually drawn in the image. Points along the borders allow manual correction of the segmentation. **b** Resulting strain curves for LV circumferential (dashed line) and radial strain (solid line). Different markers along the curves (dot and asterisk) correspond to different timepoints in the cine image sequence, i.e., there are 30 temporal phases.

Feature tracking was performed retrospectively on short-axis and long-axis cine images in left and right ventricle using Segment software v.2.0 R5585 [22, 23]. The software uses a non-rigid elastic registration scheme with limited-memory optimizer to track tissue borders in each cine image frame. The feature tracking analysis was performed by manually drawing the wall contours in the first cine image at the end of diastole (Fig. 1a). The rest of the image sequence was segmented automatically by the software and corrected manually if needed.

As a result, time–strain curves covering the entire cine image sequences, were obtained (Fig. 1b). Mean circumferential strain curves were derived in short-axis direction from basal, mid ventricular, and apical cine images, and mean longitudinal strain curves from four-chamber cine images. The curves were used to calculate peak systolic strain (%), peak systolic strain rate (%/s), and peak diastolic strain rate (%/s) in both ventricles in circumferential and longitudinal directions. Strain values were calculated using MATLAB R2016A (The MathWorks, Inc. Natick, MA, USA). Nine subjects (four controls, five TOF patients) had no four-chamber cine images and thus no longitudinal strain parameters were obtained in them.

Ventricular dyssynchrony in TOF has been well established, and RV contraction has been shown to be delayed in TOF patients compared to healthy controls [19, 24]. This makes the comparison of the strain curve of a TOF patient and a healthy control troublesome. We wanted to visually compare the strain curves, and thus used heart rate correction for the RR interval. After calculating the strain values, the RR interval of all strain curves was linearly corrected to match the mean heart rate of healthy controls (heart rate of 68), to allow visual comparison between healthy controls and TOF patients. The correction is analogous to Bazett’s formula for heart rate corrected QT interval. The reason for this correction was that heart rate determines the length of the RR-interval, and without heart rate correction no visual comparison between the strain curves is possible.

### Statistical analysis

Statistical analysis was performed using IBM SPSS Statistics for Windows 24 (IBM Corp., Armonk, NY, USA). Results are reported as mean ± standard deviation. Comparison of continuous data between the groups was performed using independent samples *t *test. No equal variances were assumed. Correlations were calculated using Pearson correlation coefficient. Two-sided tests were used and *p *values of less than 0.05 were considered statistically significant.

## Results

Characteristics of the study population and control subjects are presented in Table 1. As pulmonary regurgitation is often held responsible for many of the late complications in TOF, we were especially interested in whether the severity of PR would affect RV and LV strain. Hence, the study population was divided into two using 30 ml/m^2^ of PR as a cut-off limit for severe pulmonary regurgitation [25].

### Strain findings

RV peak longitudinal strain was significantly increased in TOF patients with PR > 30 ml/m^2^ when compared to those with PR < 30 ml/m^2^ (− 22.5% ± 2.7% vs − 19.7% ± 3.5%, *p* = 0.018) and controls (*p* = 0.007, Table 2). There was no difference in RV peak longitudinal strain between TOF patients with PR < 30 ml/m^2^ and controls. Within TOF patients, correlations were found between PR volume and peak RV longitudinal strain (*R* = − 0.37, *p* = 0.030), PR volume and peak RV systolic longitudinal strain rate (*R* = − 0.37, *p* = 0.030), and PR volume and peak RV diastolic longitudinal strain rate (*R* = 0.39, *p* = 0.021). Thus, patients with florid pulmonary regurgitation had, on average, higher longitudinal peak strain, and systolic and diastolic strain rate in the TOF population. Similar findings were demonstrated when the postoperative study population was grouped in two according to the presence of transannular patch (TAP, data not shown). Since the use of TAP was not always determinant for severe PR, we decided to maintain PR volume of 30 ml/m^2^ as a cut-off value to divide the TOF patients [25].

**Table 2:** Results for right ventricle strain parameters of healthy controls and of patients with TOF having pulmonary regurgitation

Parameter	Healthy	TOF	*p* value	TOF (PR < 30 ml/m^2^)	TOF (PR > 30/m^2^)	*p* value
RV circumferential	*N* = 44	*N* = 40		*N* = 29	*N* = 11	
Strain (%)	− 11.5 ± 3.2	− 16.6 ± 3.9	< 0.0001*	− 16.7 ± 3.6	− 17.0 ± 4.1	0.698
Systolic strain rate (%/s)	− 59.9 ± 20.9	− 79.8 ± 23.4	< 0.0001*	− 77.9 ± 21.8	− 84.8 ± 32.1	0.472
Diastolic strain rate (%/s)	60.8 ± 19.2	87.0 ± 33.0	< 0.0001*	84.4 ± 32.1	93.9 ± 35.7	0.447
RV longitudinal	*N* = 40	*N* = 35		*N* = 24	*N* = 11	
Strain (%)	− 19.4 ± 3.2	− 20.6 ± 3.5	0.147	− 19.7 ± 3.5	− 22.5 ± 2.9	0.018*
Systolic strain rate (%/s)	− 83.5 ± 18.5	− 99.3 ± 23.1	0.002*	− 94.7 ± 20.2	− 109.4 ± 26.6	0.122
Diastolic strain rate (%/s)	81.1 ± 26.9	106.7 ± 32.8	0.0001*	101.5 ± 27.1	124.4 ± 39.6	0.103

*PR* pulmonary regurgitation, *RV* right ventricle, *TOF* repaired tetralogy of Fallot

*Statistically significant difference (*p* < 0.05)

**Fig. 2:**
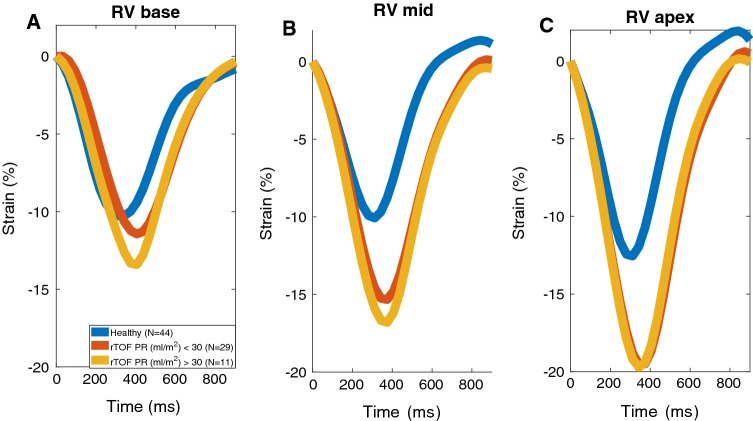
Mean RV circumferential strain curves in basal, mid-ventricular, and apical planes with heart rate corrected to 68 (mean HR of healthy controls) in each group. *RV* right ventricle, *rTOF* repaired tetralogy of Fallot, *PR* pulmonary regurgitation

When looking at RV circumferential strain in different short-axis planes (Table 3), peak strain was significantly increased in all planes in the entire TOF population. The increase is highest at the mid-ventricular and apical levels (*p* < 0.0001). There were no differences between the two TOF subgroups. The same finding was seen in the heart-rate fixed strain curves of different RV short-axis planes (Fig. 2): the RV circumferential strain increased gradually from base to apex in all study groups. However, the increase of peak strain towards the apex was stronger in patients with TOF (apex-base difference 7.6% ± 4.2% vs 3.3% ± 2.4%, *p* < 0.0001) in whom peak basal, mid-level, and apical strain were all significantly different from each other. In healthy controls the basal and mid-level strain remained similar. However, PR did not correlate with different RV circumferential strain components in TOF patients.

**Table 3:** Peak circumferential strain in three planes of the right ventricle of healthy controls and of patients with TOF having pulmonary regurgitation

RV plane	Healthy (*N* = 44)	TOF (*N* = 40)	*p* value	TOF PR < 30 (*N* = 29)	TOF PR > 30 (*N* = 11)	*p* value
Base	− 11.2 ± 2.9	− 13.1 ± 3.8	0.01*	− 12.8 ± 4.0	− 13.9 ± 3.1	0.341
Mid	− 10.7 ± 3.4	− 16.8 ± 4.2	< 0.0001*	− 16.6 ± 4.0	− 17.3 ± 5.0	0.636
Apex	− 13.5 ± 4.2	− 20.7 ± 5.0	< 0.0001*	− 20.8 ± 4.7	− 20.4 ± 6.0	0.876

*RV* right ventricle, *TOF* repaired tetralogy of Fallot, *PR* pulmonary regurgitation ml/m^2^

*Statistically significant difference (*p* value < 0.05)

**Fig. 3:**
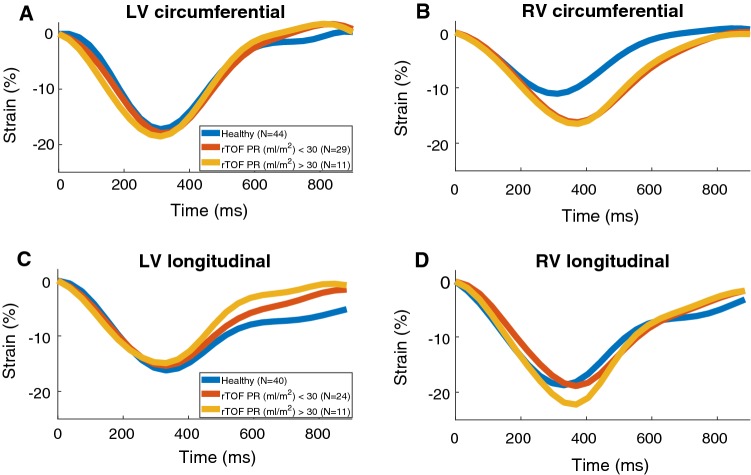
Mean strain curves with heart rate corrected to 68 (mean HR of healthy controls) in each group. *LV* left ventricle, *RV* right ventricle, *rTOF* repaired tetralogy of Fallot, *PR* pulmonary regurgitation

The heart rate fixed strain curves for RV and LV (Fig. 3) show that the timing of RV peak systolic strain occurred later in both circumferential and longitudinal directions in TOF patients when compared to healthy controls. Trajectories of RV circumferential strain were identical in the TOF groups. In the left ventricle, no difference in timing of peak strain could be seen between TOF patients and healthy controls. Peak longitudinal and circumferential strain coincided within the ventricles in all study subjects. However, our method of heart rate correction allowed demonstration of the peak circumferential and longitudinal strain occurring 50 ± 15 ms later in RV compared to LV (Fig. 3). Despite LV longitudinal strain being similar in systole, peak diastolic longitudinal LV strain rate was increased in TOF patients when compared to healthy controls (81.9%/s ± 31.1%/s vs 65.9%/s ± 19.1%/s, *p* = 0.011). The diastolic LV longitudinal strain was highest in the TOF group with PR > 30 ml/m^2^ (Table 4). Significant correlations between EF and strain (circumferential and longitudinal) were found both in healthy controls and TOF patients, in both ventricles (*R* < − 0.37, *p* < 0.05).

**Table 4:** Results for left ventricle strain parameters in healthy controls and in patients with TOF having pulmonary regurgitation

Parameter	Healthy	TOF	*p* value	TOF (PR < 30 ml/m^2^)	TOF (PR > 30 ml/m^2^)	*p* value
LV circumferential	*N* = 44	*N* = 40		*N* = 29	*N* = 11	
Strain (%)	− 18.2 ± 2.6	− 18.6 ± 3.0	0.510	− 18.4 ± 2.9	− 19.3 ± 3.1	0.419
Systolic strain rate (%/s)	− 104.2 ± 21.6	− 112.3 ± 30.0	0.161	− 110. 3 ± 29.9	− 117.6 ± 29.1	0.488
Diastolic strain rate (%/s)	117.1 ± 28.6	124.4 ± 36.8	0.314	121.6 ± 35.4	131.9 ± 41.0	0.473
LV longitudinal	*N* = 40	*N* = 35		*N* = 24	*N* = 11	
Strain (%)	− 16.8 ± 2.7	− 15.6 ± 2.9	0.062	− 15.7 ± 3.3	− 15.2 ± 2.0	0.558
Systolic strain rate (%/s)	− 79.6 ± 17.5	− 86.6 ± 15.8	0.075	− 85.1 ± 16.1	− 89.7 ± 15.5	0.432
Diastolic strain rate (%/s)	65.9 ± 19.1	81.9 ± 31.1	0.011*	76.6 ± 24.6	93.5 ± 41.1	0.229

*PR* pulmonary regurgitation, *LV* left ventricle, *TOF* repaired tetralogy of Fallot

*Statistically significant difference (*p* < 0.05)

### Intra observer variability of strain measurements

Variability between strain measurements was evaluated using intraclass correlation coefficient (ICC). Thirty studies were reanalyzed for peak RV and LV circumferential strain and RV and LV longitudinal strain. The ICC of different strain parameters was: RV peak circumferential strain: 0.985 (0.97–0.99); RV peak longitudinal strain: 0.967 (0.93–0.99); LV peak circumferential strain: 0.976 (0.90–0.99); and LV peak longitudinal strain: 0.970 (0.94–0.99).

## Discussion

In this study, we measured longitudinal and circumferential strain in RV and LV with CMR in pediatric and adolescents with TOF. Rather than only studying peak systolic or diastolic values we additionally determined the heart rate-corrected mean strain curves, to allow visual comparison between the patients and controls. Our study also included a control population that consisted of healthy subjects with no diagnosed or suspected heart or other health issues. CMR FT offers many possible approaches in the study of TOF. We hypothesized that since the basal and outlet regions of the RV are injured at repair, possible compensatory changes in myocardial function may take place toward the apical regions.

Patients with severe PR (> 30 ml/m^2^) and increased RV diastolic volume load had higher longitudinal strain when compared with healthy controls or patients with milder PR. Patients whose PR exceeded 30 ml/m^2^ demonstrated a higher RV circumferential strain in addition to a higher RV longitudinal strain. This may reflect appropriate compensation of the right ventricular function, as strain is a measure for the deformation of the cardiac muscle tissue. However, and contrary to the present observations, in studies on adult TOF patients, no increase in RV strain related to PR has been found [24, 26]. Thus, despite perhaps being a compensatory mechanism, an initial increase in RV strain during childhood may be temporary.

Right ventricular outflow tract RVOT and ventricular septal defect area areas affected during corrective surgery, regardless of the surgical method. Superficial circumferential muscle fibers lie in parallel to these areas and are more likely to be injured than longitudinal fibers. By dividing the circumferential strain measurements to apical, mid, and basal regions, we noticed that the strain values increased from base to apex in all study groups. Strain in our normal controls was similar at basal and mid-level, and highest (on average 2.5% higher) in the apical plane, as described previously [27]. These cardiac planes functioned more uniformly in healthy individuals, while the increase in peak strain was significantly higher in mid-level and apical planes in the TOF subgroups. It is thus possible that the apical part of the chamber left untouched during surgery could compensate for the reduced strain observed within regions subjected to injury. Anwar et al. reported diminished longitudinal strain of the RVOT in TOF patients repaired with TAP when compared to pulmonary stenosis patients who had undergone balloon valvuloplasty [28]. They proposed that the non-contractile transannular patch could explain this difference. However, our observation of increased RV strain in TOF patients further suggests that especially the mid and apical regions undergo a deformation process that serve as a compensation mechanism.

The results for the patients presented here demonstrate that after approximately 10 years of TOF-repair, timing of both circumferential and longitudinal RV peak systolic strain when compared to the controls was delayed. This observation is consistent with the right bundle branch block observed commonly after TOF-repair. LV longitudinal strain in diastole returned to zero faster in patients with TOF than in healthy subjects. It has been previously documented that left ventricular preload is decreased in TOF patients and is caused by decreased transpulmonary flow due to pulmonary regurgitation [17]. It is possible that the diastolic phase of our patients was affected by the smaller stroke volume. Similar to the observation on the timing of LV longitudinal strain in diastole, we observed no differences in LV strain values between patients and controls or between the two patient groups, although a small but significant difference in LV ejection fraction was noted. It seems that, as opposed to the right ventricle, the left ventricle had remained undisturbed in our young postoperative patients with TOF. However, as reported previously, LV myocardial abnormalities might develop later in adulthood. We conjecture that the observed slight delay in peak strain between RV and LV may be an indication of disturbed ventricular interaction. It should be interesting to perform similar measurements in older patients of TOF and heart failure due to left ventricular dysfunction**.**

Diastolic strain parameters have been difficult to assess with CMR tagging, the gold standard in myocardial motion analysis, due to dephasing of magnetization. Hence, we chose to assess the myocardial function with CMR FT, where the image quality is maintained throughout the cardiac cycle. Recently, strain results with CMR FT were shown to have inter-software variability in both feasibility and absolute strain values [29]. However, the non-rigid elastic registration-based FT used in this work has been shown to be reproducible and not be influenced by the level of training [30]. Our intra observer variability analysis agreed with this result.

### Limitations

PR was calculated using ventricular volumetric data and not phase-contrast angiography, this is a limitation in our study. The reported *R* values are rather small to be clinically useful, and the number of patients with severe pulmonary regurgitation was relatively small compared to other groups.

## Conclusion

RV longitudinal strain is increased in pediatric TOF patients with severe pulmonary regurgitation. In comparison to healthy volunteers, all TOF patients demonstrate enhanced right ventricular circumferential strain, accentuated in the apical region. Instead, left ventricular myocardial function, as assessed by strain, is well preserved in children and adolescents with contemporary postoperative TOF. CMR FT analysis of strain offers an additional tool to assess RV function in TOF, a clear benefit is that the present method can be applied retrospectively to images obtained from individuals studied by conventional MRI protocols. Finally, our data on healthy children and adolescents can be used as a reference when assessing different congenital heart defects with CMR FT.
